# A Case of Trastuzumab-Associated Cardiomyopathy Presenting as an Acute Coronary Syndrome: Acute Trastuzumab Cardiotoxicity

**DOI:** 10.1155/2013/473979

**Published:** 2013-03-06

**Authors:** Sylvana Hidalgo, Carol A. Albright, Gretchen L. Wells

**Affiliations:** ^1^Department of Internal Medicine, Section on Cardiology, Wake Forest School of Medicine, Medical Center Boulevard, Winston-Salem, NC 27157-1045, USA; ^2^Department of Internal Medicine, Section on Hematology and Oncology, Wake Forest School of Medicine, Medical Center Boulevard, Winston-Salem, NC 27157-1045, USA

## Abstract

Trastuzumab is a monoclonal antibody highly effective in the treatment of several cancers, but its use is associated with cardiac toxicity which usually responds to cessation of the drug and/or medical therapy. We present an unusual case of acute cardiac toxicity temporally related to administration of trastuzumab in which the clinical presentation suggested an acute coronary syndrome. Coronary angiography, however, demonstrated minimal epicardial disease, but new wall motion abnormalities. Furthermore, the patient did not respond to withdrawal of the drug or medical therapy for heart failure.

## 1. Introduction 

Trastuzumab is a monoclonal antibody part of standard medical therapy for both early and advanced breast cancer in individuals whose tumors overexpress the human epidermal growth factor receptor 2 (HER2) protein [1]. HER2 is overexpressed in other tumor types as well, including gastric, endometrial, and lung. Its overexpression portends a poor prognosis [2]. Trastuzumab significantly improves both disease-free survival and overall survival among women with HER2-positive breast cancer [1, 3].

Cardiotoxicity has been reported to occur with trastuzumab when administered alone and in combination with antineoplastic agents, particularly anthracyclines [4]. The risk of cardiotoxicity with trastuzumab has been reported to be 4% with monotherapy and 27% when administered in combination with an anthracycline and cyclophosphamide. However, clinical trials of trastuzumab have typically enrolled younger women without cardiac comorbidities who do not represent most of the women treated for breast cancer. Analysis of the Surveillance, Epidemiology, and End Results-(SEER-) Medicare-linked database found an absolute 14% higher adjusted incidence of cardiac complications (heart failure or cardiomyopathy) in older women (mean age 76 years) undergoing treatment with trastuzumab and an absolute 23.8% higher rate in those undergoing treatment with both trastuzumab and anthracycline for breast cancer [5]. Therefore, the incidence of cardiotoxicity is much higher in a “real-world” analysis than previously published in clinical trials. Importantly, unlike anthracycline-induced toxicity, trastuzumab-associated toxicity usually responds to standard heart failure treatment or the discontinuation of trastuzumab, and there is no evidence that the toxicity is dose related [6].

## 2. Case Presentation 

We report a case of trastuzumab-associated cardiotoxicity, initially manifesting as an acute coronary syndrome (ACS), in a 59-year-old male with metastatic adenocarcinoma of the esophagus. The patient was diagnosed in October 2010 (20 months prior to his ACS admission). His initial treatment involved cisplatin, 5-fluorouracil, and radiation followed by esophagogastrectomy and bilateral myotomy. Progression of his disease was noted the following year in April 2012. He was initiated on FOLFOX (folinic acid, fluorouracil, and oxaliplatin) and trastuzumab for HER2-positive disease. The FOLFOX was given every two weeks and the trastuzumab every three weeks.

**Figure 1:**
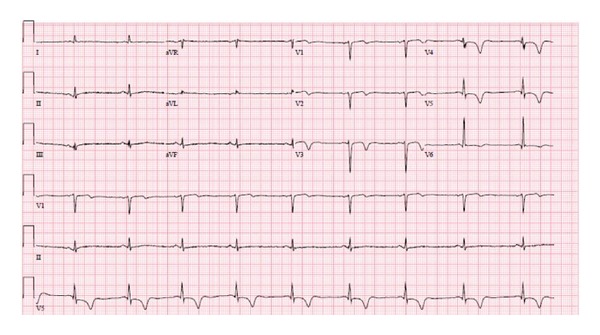
12-lead ECG at presentation demonstrating new anterolateral T-wave inversions.

**Figure 2:**
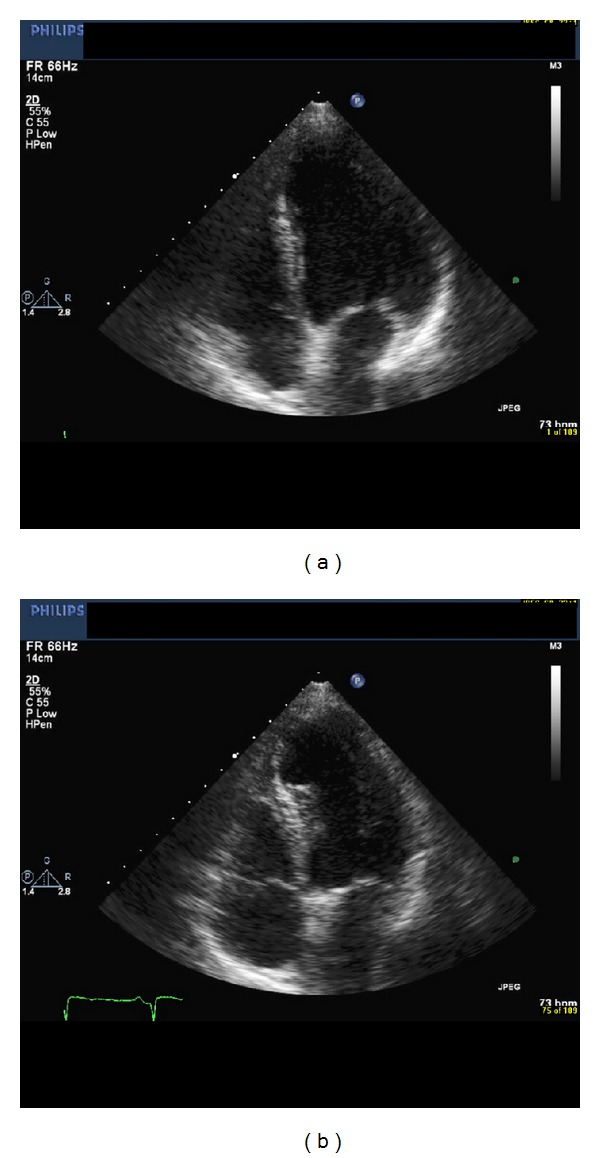
Two-dimensional echocardiographic view demonstrating new anterior wall motion abnormality (apical 4-chamber view) at diastole (a) and systole (b).

Immediately following his third trastuzumab infusion, he developed chest tightness, shortness of breath, and nausea. An EKG demonstrated new anterolateral T-wave inversions (Figure 1). Significant laboratory data included an initial troponin I of <0.006 ng/mL (normal 0.000–0.040 ng/mL) and a hemoglobin of 11.7 g/dL. He was transferred to a tertiary level medical center coronary care unit where his cardiac biomarkers remained negative (peak troponin I 0.007 ng/mL). An echocardiogram demonstrated new anterior and anteroseptal wall motion abnormalities (Figure 2) and an ejection fraction of 40%. He was begun on standard cardiac medications, including a beta blocker. A cardiac catheterization demonstrated mild nonobstructive coronary artery disease with wall motion abnormalities on the left ventriculogram, consistent with findings on the echocardiogram. The decision was made to hold treatment with trastuzumab until his left ventricular function improved. However, followup echocardiograms (four and ten weeks after cardiac hospitalization) demonstrated persistent left ventricular dysfunction with ejection fractions of 40%. Of note, serial echocardiograms prior to and during chemotherapy all demonstrated normal left ventricular systolic function.

## 3. Discussion 

This case report is unique in that we describe a male undergoing chemotherapy with trastuzumab who presents with findings mimicking an acute coronary syndrome. Thus far, other similar case reports have described only women [7, 8]. These women presented with transient left bundle branch block, and their left ventricular function improved with standard heart failure medical therapy and discontinuation of drug. In our patient, the left ventricular function did not improve with medical therapy and cessation of trastuzumab.

The pathogenesis of trastuzumab-associated cardiotoxicity is not completely understood. Unlike anthracycline cardiotoxicity where cardiac damage is believed to result from free radical injury and oxidative stress resulting in myocyte loss, trastuzumab cardiotoxicity is believed to result from blocked ErbB2 signaling pathways. The proto-oncogene ErbB2 (also known as c-Neu or HER2 in humans) encodes a receptor tyrosine kinase critical in cardiovascular development. Genetically manipulated mice lacking ErbB2 die in utero with failure of cardiac development. In the postnatal heart, ErbB2 conditional knockout mice develop dilated cardiomyopathy at eight weeks of life. How this occurs at the molecular level remains under active investigation. Expression of ErbB receptors decreases in chronic heart failure in humans, suggesting that altered ErbB signaling plays a role in the progression of heart failure [9].

Important lessons emerge from this unique case report. First, we describe a case of trastuzumab cardiotoxicity mimicking ACS presenting in a man, which is different from those previously described in women. Numerous studies have reported a difference in heart failure between men and women, thus suggesting that variable expression of myocyte receptors (or ligands) in men and women could be involved in the development and progression of heart failure. Unlike other studies of trastuzumab cardiotoxicity, this patient appears to have had irreversible cardiac damage despite minimal epicardial disease, standard heart failure treatment, and cessation of the drug. Clinicians should be aware of this possibility when caring for patients with trastuzumab-associated cardiotoxicity.
